# Mechanical, Thermomechanical and Reprocessing Behavior of Green Composites from Biodegradable Polymer and Wood Flour

**DOI:** 10.3390/ma8115406

**Published:** 2015-11-11

**Authors:** Marco Morreale, Antonio Liga, Maria Chiara Mistretta, Laura Ascione, Francesco Paolo La Mantia

**Affiliations:** 1 Facoltà di Ingegneria e Architettura, Università degli studi di Enna “Kore”, Cittadella Universitaria, Enna 94100, Italy; 2 Institute of Biological Chemistry, Biophysics and Bioengineering, Heriot-Watt University, Edinburgh EH14 4AS, UK; al4@hw.ac.uk; 3 Dipartimento di Ingegneria Civile, Ambientale, Aerospaziale, dei Materiali, Università di Palermo, Viale delle Scienze, Palermo 90128, Italy; mariachiara.mistretta@unipa.it (M.C.M.); laura.ascione@unipa.it (L.A.); francescopaolo.lamantia@unipa.it (F.P.L.M.)

**Keywords:** green composites, biopolymer, mechanical properties, dynamic mechanical analysis, creep, thermal analysis

## Abstract

The rising concerns in terms of environmental protection and the search for more versatile polymer-based materials have led to an increasing interest in the use of polymer composites filled with natural organic fillers (biodegradable and/or coming from renewable resources) as a replacement for traditional mineral inorganic fillers. At the same time, the recycling of polymers is still of fundamental importance in order to optimize the utilization of available resources, reducing the environmental impact related to the life cycle of polymer-based items. Green composites from biopolymer matrix and wood flour were prepared and the investigation focused on several issues, such as the effect of reprocessing on the matrix properties, wood flour loading effects on virgin and reprocessed biopolymer, and wood flour effects on material reprocessability. Tensile, Dynamic-mechanical thermal (DMTA), differential scanning calorimetry (DSC) and creep tests were performed, pointing out that wood flour leads to an improvement of rigidity and creep resistance in comparison to the pristine polymer, without compromising other properties such as the tensile strength. The biopolymer also showed a good resistance to multiple reprocessing; the latter even allowed for improving some properties of the obtained green composites.

## 1. Introduction

Rising concerns regarding environmental issues, public health, and stricter legislations are currently driving research attention on the development of biomaterials coming from renewable sources. Although in the last decades plastic recycling experienced a large growth in capacity and technology, the fraction of plastics waste annually disposed in landfills is still the most significant [1]. Furthermore, most of the common polymeric materials are highly resistant to physical and microbial degradation and become environmental polluters [2]. The packaging industry involves huge quantities of plastics and since it deals often with biodegradable products, such as food, this would suggest the use of biodegradable polymers [3].

Biopolymers such as poly(lactic acid) (PLA) and Mater-Bi present mechanical behavior similar to petroleum-based materials and are easily compostable, due to their high biodegradation rate and nontoxicity. On the other hand, these materials can be too expensive if compared to traditional plastics [4]. An effective way to reduce cost, without affecting environmentally friendly behavior, is the development of green composites from biopolymers and natural fillers. Composites are largely employed in several industrial sectors, especially in transport, and since their recycling is often not economically feasible, this results in a growing rate of waste accumulation [5]. For these reasons, the development of compostable or easily recyclable composites would be of great interest for plastics sustainability. In recent years, several studies investigated the opportunity to replace traditional fillers, such as glass fibers or carbon black, with natural based materials [6]. Fillers can be derived from different sources including starch [7], chitin, microcrystalline cellulose [6], rice husk [8], jute [9], wood flour [10], cassava, starch-green coconut [11], palm [12], hemp [13], caraua [14], bagasse [4], bamboo, coffee ground [15], poultry feathers [16], flax [17], sisal, and cotton [18]. The benefit of using natural based materials lies, among other reasons, on reducing dependency on oil [19]. Of course, for the biomaterial to be economically convenient fillers need to be low-cost and available in huge quantities; therefore, research is focusing on food/agricultural industry waste products. Using natural fibers as fillers, beside renewability and biodegradability, can also result in an easier processing, and subsequent energy saving [20]. Additionally, in some authors’ opinion, energy recovery from filled-biopolymers incineration can be seen as a contribution to renewable energy production [21].

Green composites are usually lightweight, fully sustainable materials. Nevertheless they still generate some concerns, mostly caused by economic reasons, their limited shelf life, and their mechanical properties, often worse than those of petroleum-based materials. Furthermore there are concerns that compostable bio-plastics will upset existing recycling methods [19]. Most biopolymers were originally designed for packaging, so they are not optimized to form the matrix in a composite. Matrices will be chosen according to mechanical, chemical, and thermal requirements of the final object and should have low viscosity, so as to guarantee the complete impregnation of fibers [22]. In some cases matrices need to be modified in order to enhance the matrix-fibers surface adhesion; valid alternatives to matrix treatment can be the use of coupling agents or fiber treatment in order to improve natural fiber/matrix interfacial adhesion [23,24,25,26]. Nevertheless, all solutions lead to additional costs and to the possibility of reducing the biodegradability.

Natural fibers can also be used to reevaluate recycled matrices with compromised mechanical properties. In particular when testing high-density polyethylene (HDPE)-hemp composites it was shown that the matrix-fibers interactions, using a recycled matrix, were stronger than using virgin material [13]. Other studies showed how the presence of fibers can modify the reprocessability of materials. When the affinity of matrix-fibers is high, reprocessing can improve the fibers dispersion and surface area, thus resulting in improved recyclability. Otherwise, if the affinity of matrix-fibers is low, fibers behave more as a filler than as a reinforcement, and material recyclability decreases with fiber content [27]. It seems clear how it can be complicated to foresee the effect of the introduction of fibers in the same material, at different reprocessing stages, and the effect of fibers on degradation phenomena.

BioFlex F2110 is a new generation of commercial biopolymer produced by FKuR Kunststoff GmbH (Willich, Germany), based on blend of PLA and Thermoplastic-Copolyesters (TPC) [28], and in spite of its interesting mechanical properties, to the best of our knowledge, no example of its characteristics as a matrix in fiber-reinforced materials can be found in scientific literature. Testing on wood flour/fiber (WF) content effects on some properties of a different version of BioFlex, the 467-F (consisting primarily of renewable resources and biodegradable polymers) was performed by Sykacek *et al.* [29] stating improvements in tensile and flexural strength and modulus at the expense of lower impact strength and higher water uptake. Other studies on WF-natural based polymer composites showed slightly different trends, reporting a constant tensile strength with growing fiber content, but similar behavior for the other parameters [30].

This paper focuses on the characterization of BioFlex F2110-wood flour green composites, with the addition of fibers at different recycling stages. After loading the fibers, composites were further processed, by means of a single screw extrusion followed by compression molding. This way, it was possible to get a better understanding of the reprocessing effect on biopolymer matrix properties, wood flour loading effects on virgin and reprocessed biopolymer, and wood flour effects on material reprocessability.

## 2. Results and Discussion

Tensile characterization allowed for the comparison of the elastic modulus, tensile strength, and elongation at break of the different systems. Dynamic-mechanical thermal (DMTA) analysis was mainly important to investigate the complex modulus components, the glass transition temperatures, and their relationship with the thermomechanical resistance of the different systems. Given the high number of specimens analyzed (as described in the Experimental section), and their mutual connections, results and discussion is organized according to individual manufacturing parameters.

### 2.1. Recycling on Pristine BioFlex

Processing the neat polymer in a single screw extruder up to four times did not lead to significant reductions in the elastic modulus (Table 1). This applies also to the tensile strength. On the other hand, a slightly lower elongation at break (still statistically significant, *p* = 0.0251) was observed, which could imply the shortening of polymer chains during the reprocessing.

DMTA tests showed the trends for the components of the complex modulus, *i.e.*, storage (*E*′) and loss (*E*′′) moduli taken at 55 °C (See Table 2). In particular, *E*′ showed only minor variations (in agreement with the minor variations observed with reference to the elastic modulus), while the glass temperature, *T*_g_, experienced a slight reduction. This seems to indicate a moderate shortening of polymer chains, resulting in a slightly higher mobility, and is in agreement with previous studies on recycling of PLA and other polyesters [31,32,33].

**Table 1:** Elastic modulus (*E*), tensile strength (TS), elongation at break (EB) for pristine (V) and reprocessed (two times: R2, four times: R4) BioFlex.

Property	V	R2	R4
***E* [MPa]**	142	141	143
**TS [MPa]**	10.7	10.7	10.1
**EB [%]**	123	119	99

**Table 2:** Dynamic-mechanical thermal (DMTA) results for pristine and reprocessed BioFlex.

Property	V	R4
***E*′ [MPa]**	120	124
***E*′′ [MPa]**	25.7	13.1
***T*_g_ [°C]**	65	63

### 2.2. Wood Flour Content

Tensile tests showed an increasing trend for the elastic modulus of BioFlex with increasing amounts of filler. As shown in Table 3, samples containing 15% and 30% wood flour (*i.e.*, V15 and V30) presented elastic moduli respectively 220% and 380% higher than the original BioFlex (V). Besides the increasing in elastic modulus, wood flour addition determines a drop of the elongation at break (mainly due to the stiffening effect of the filler), while tensile strength does not change much upon changing the wood flour content (15% or 30%). This is in adequate agreement with the results found in other works dealing with natural organic fillers (in particle form) and other biodegradable polymers [30,34,35,36,37] and could be mainly attributed to a decent degree of adhesion between the polar natural organic filler and the biopolymer. On the other hand, when apolar polymer matrices (such as polyolefins) are involved, the increase in filler content usually leads to a decrease in the tensile strength [38].

**Table 3:** Elastic modulus (*E*), tensile strength at break (TS), elongation at break (EB) for neat polymer (V), four-times reprocessed polymer (R4), 15% and 30% filled composites (V15, V30), 15% and 30% filled composites prepared after multiple recycling of the polymer matrix (R4-15, R4-30).

Property	V	R4	V-15	V-30	R4-15	R4-30
***E* [MPa]**	142	143	313	539	317	511
**TS [MPa]**	10.7	10.1	9.2	11.2	10.3	11.6
**EB [%]**	123	99	12.9	6.6	11.4	6.8

Similar considerations can be drawn also for reprocessed BioFlex (R4) after wood flour addition (R4-15, R4-30): a remarkable increase of the rigidity is observed, whereas variations of the tensile strength are only marginal. On the other hand, elongation at break drastically decreases, as previously observed. Therefore, adding wood flour can be a simple, cost-saving and effective way to increase some of the mechanical properties of recycled biodegradable polymers such as BioFlex, in applications where high ductility levels are not required.

**Figure 1:**
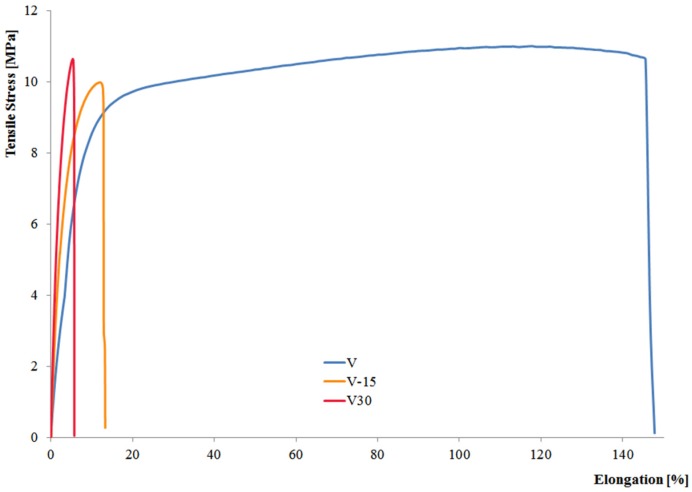
Comparison on stress-strain curves for virgin BioFlex at different wood flour content.

In Figure 1, stress-strain curves are shown for samples of the neat pristine matrix (V) and composites loaded with 15% (V15) and 30% (V30) wood flour. The dramatic reduction in elongation at break (and thus in ductility) and, at the same time, the significant increase in elastic modulus (*i.e.*, in rigidity) can be easily observed.

Dynamic mechanical analysis (Table 4) gave, for E′ and E′′ (at 55 °C), the same trends showed by tensile testing for elastic modulus. Furthermore, it was shown that *T*_g_ increases with WF content. This is probably due to steric hindering caused by wood fibers, which prevents macromolecular chain movement, and is in agreement with the findings of other researchers. Altun *et al.* [39] reported that the glass transition temperature of PLA-wood fiber composites shifted to slightly higher temperatures, due to the restricted molecular motion arising from strong interaction between the matrix and filler. Similar trends were also found by Huda *et al.* [40].

**Figure 2:**
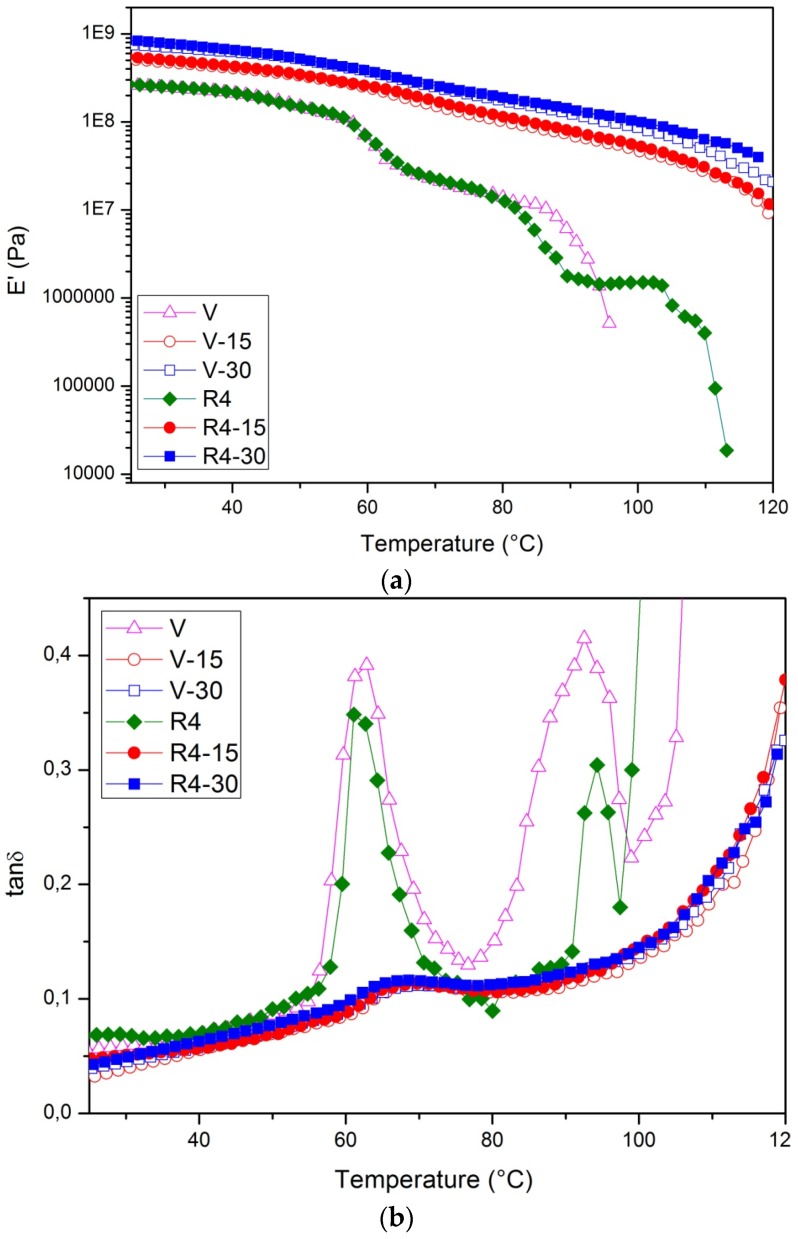
Storage modulus (**a**) and damping factor (**b**) for the investigated samples (V = pristine polymer, R4 = four-times reprocessed polymer, V15 and V30 = 15% and 30% filled composites, R4-15 and R4-30 = 15% and 30% filled composites prepared with polymer that has been reprocessed four times).

Figure 2a,b reports, for the entire temperature range of investigation, the DMTA curves for the storage modulus and the damping factor, respectively. The E′ curves evidence the overall trend of the different samples, confirming that the addition of wood flour significantly increases the modulus over the entire range, while reprocessing leads only to minor variations: this suggests that only marginal shortening of the polymer chains has occurred.

**Table 4:** Comparison between DMTA results of neat polymer (V), 15% and 30% filled composites (V15, V30) and 15%, 30% filled composites prepared after multiple recyclings of the polymer matrix (R4-15, R4-30).

Property	V	R4	V-15	V-30	R4-15	R4-30
***E*′ [MPa]**	120	124	286	436	296	442
***E*′′ [MPa]**	25.7	13.1	22.1	35.1	23.4	35.7
***T*_g_ [°C]**	65	63	69	70	70	70

Regarding the other observable variations upon adding the wood flour, these are substantially small, although they are more significant (up to approximately 10%) when wood flour is added to the already reprocessed BioFlex. In all cases, the peak becomes lower and broader upon adding wood flour, thus indicating significant interaction phenomena between the matrix and the wood particles.

### 2.3. Recycling of Filled BioFlex

Tensile tests (Table 5) showed that samples recycled four times and then loaded with wood flour highly benefit from one additional reprocessing through a single screw extruder. In particular, the elastic modulus increases about 10% and 17%, when passing from R-15 and R-30 to RP-15 and RP-30 respectively. Specimens prepared with matrix polymer not recycled before filling (V-15, V-30) show much more modest differences after reprocessing (VP-15, VP-30), not always with a positive trend. Nonetheless, DMTA tests (Table 6) confirm the increase in mechanical properties for both kinds of samples (even if it is more modest for the non-recycled one). This phenomenon can be explained by invoking a higher degree of mixing, with improved surface area between wood fibers and polymer chains, possibly further enhanced in the presence of moderately shorter polymer chains (like in previously recycled polymers). This makes the transfer of mechanical stress easier, thus increasing the elastic modulus. Regarding the *T*_g_, this also experiences a very moderate increase, in agreement with the already discussed trends of the mechanical properties.

**Table 5:** Elastic modulus (*E*), tensile strength at break (TS), elongation at break (EB) for all of the samples (neat polymer: V; four-times reprocessed polymer: R4; 15% and 30% filled composites: V15 and V30; 15% and 30% filled composites prepared after repeated recycling of the polymer matrix: R4-15 and R4-30; 15% and 30% filled composites subjected to an additional processing step: VP-15, VP-30; 15% and 30% filled composites prepared after repeated recycling of the polymer and subjected to additional processing step: RP-15, RP-30).

Property	V	R4	V-15	V-30	R4-15	R4-30	VP-15	VP-30	RP4-15	RP4-30
***E* [MPa]**	142	143	313	539	317	511	300	477	381	565
**TS [MPa]**	10.7	10.1	9.2	11.2	10.3	11.6	8.9	10.8	10.6	11.2
**EB [%]**	123	99	12.9	6.6	11.4	6.8	11.5	7.3	10.3	5.5

**Table 6:** DMTA results for all of the samples (abbreviations as in Table 5).

Property	V	R4	V-15	V-30	R4-15	R4-30	VP-15	VP-30	RP4-15	RP4-30
***E*′ [MPa]**	120	124	286	436	296	442	296	487	333	512
***E*′′ [MPa]**	25.7	13.1	22.1	35.1	23.4	35.7	25.2	43.5	3.12	4.58
***T*_g_ [°C]**	65	63	69	70	70	70	69	71	70	71

**Figure 3:**
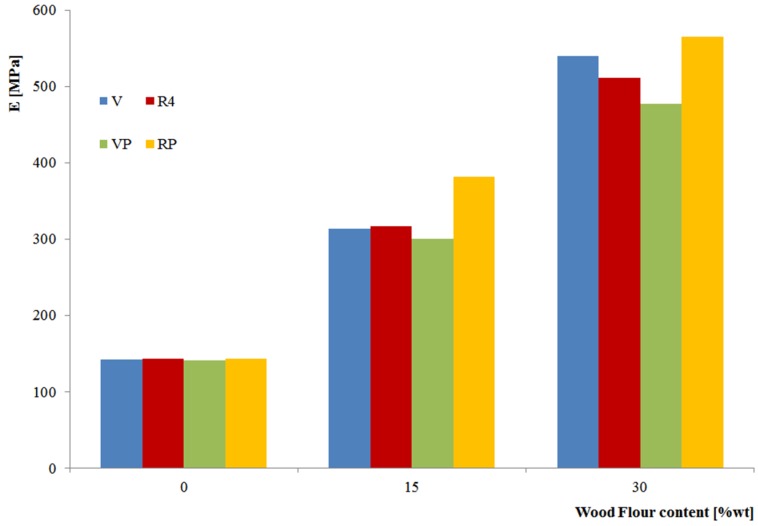
Elastic modulus of pristine BioFlex and related composites (abbreviations as in Table 5).

Figure 3 offers a direct and simultaneous comparison of the above described effects (wood flour content, recycling on neat and filled BioFlex) with regard to the elastic modulus.

### 2.4. Viscoelastic Response

**Figure 4:**
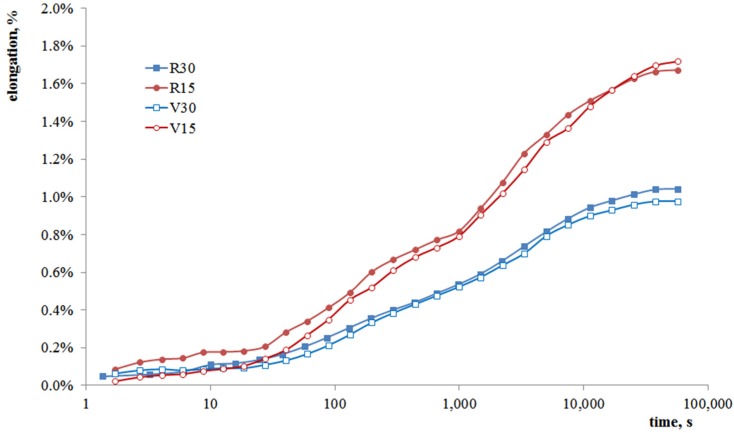
Creep curves of the composites at 60 °C and 1.5 MPa load (15% and 30% filled composites: V15 and V30; 15% and 30% filled composites prepared after repeated recycling of the polymer matrix: R15 and R30).

Figure 4 shows the creep curves (elongation *vs*. time) for the composites at 60 °C and 1.5 MPa. It can be noticed that the presence of higher wood flour percentages leads to a higher creep resistance as well, in complete agreement with the results from mechanical and DMA tests, which pointed out the increase in rigidity and thermomechanical resistance due to the wood flour. Regarding the effect of polymer matrix repeated recycling (“V” *vs.* “R” samples), the differences are practically negligible, in agreement with the behavior shown by the same materials under DMA tests.

### 2.5. Thermal Analysis

In order to deepen the investigation of the prepared systems, thermal analysis was carried out via differential scanning calorimetry. Results of second heating and cooling runs are reported in Table 7.

**Table 7:** DSC data (*T*_c_ = crystallization temperature, *T*_m_ = melting temperature, Δ*H*_c_ = crystallization enthalpy, Δ*H*_m_ = melting enthalpy) for second heating and cooling runs of all of the samples (abbreviations as in Table 5).

Sample	Cooling		Second Heating	
	T_c_ [°C]	ΔH_c_ [J/g]	T_m_ [°C]	ΔH_m_ [J/g]
V	84.5	7.5	153	6.7
V-15	86.8	8.1	152.5	7.2
V-30	87.5	8.3	151	8.3
R4	87.2	7	153	6.5
R4-15	88.6	7.5	152	7.4
R4-30	88.8	8.1	152	8.3
RP4-15	88.7	7.6	152	7.9
RP4-30	88.8	8.6	152	8.5

It can be observed that the BioFlex melting temperature slightly decreases upon increasing the WF content, thus invoking a lower quality of crystals formed in the presence of wood fibers. On the other hand, the variations are so small that the actual consequences should be considered as negligible. Regarding the melting enthalpy, the values increase upon increasing the wood flour content, in agreement with other studies on other commercial biodegradable polyester-based polymers and wood flour [41]. It is therefore hypothesized here that the crystals, even with slightly lower quality degree, are present in greater amounts, and that wood fibers act like a mild crystallization promoter in BioFlex composites.

This is confirmed also by the crystallization temperature values. Indeed, it was noticed that crystallization temperature slightly increases upon adding wood flour, thus indicating that the presence of wood fibers makes crystallization start a little in advance, when compared to unfilled polymer. Furthermore, it can be noticed that crystallization was also easier (*i.e.*, starting at a higher temperature) in the recycled material than in the one that were not reprocessed. This can be considered as further proof that recycling moderately shortens polymer chains, easing crystallization kinetics.

### 2.6. Morphology

**Figure 5:**
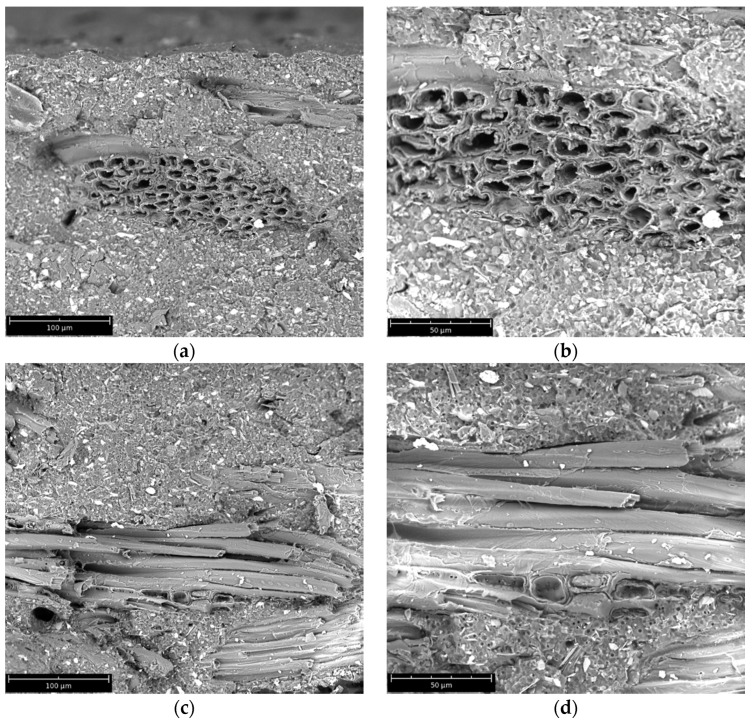

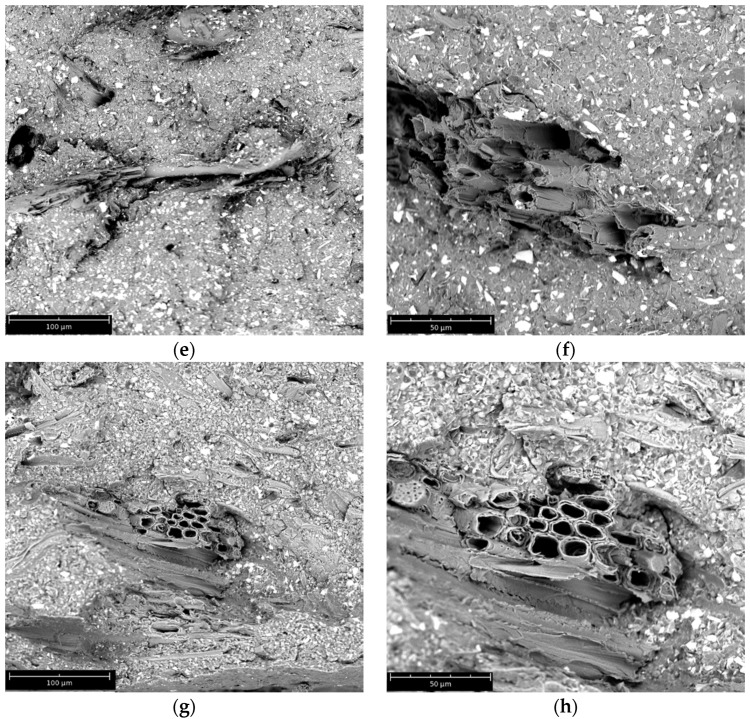
Morphology of fractured surfaces of samples (**a**,**b**) V-15; (**c**,**d**) V-30; (**e**,**f**) R4-15; (**g**,**h**) R4-30 at different magnifications.

Figure 5a–h show, respectively, the morphology of fractured surfaces of V-15 (a, b), V-30 (c, d), R4-15 (e, f), R4-30 (g, h) samples at different magnifications. These representative SEM images allow for assessing that fibers show a good adhesion with the matrix polymer, as clearly visible from the good wetting of the fibers themselves. This helps in explaining the results from tensile tests, in particular the tensile strength which did not decrease in 15 wt % filled samples and even increased in 30 wt % filled samples, whereas the trend is, in general, the complete opposite in WF-based composites where interface adhesion in unsatisfactory [36,39,42]. Furthermore, SEM analysis did not point out significant differences between the “V” and the “R” samples, a result in accordance with the small differences between the two, observed from the results of mechanical and thermomechanical characterization.

## 3. Experimental Section

### 3.1. Materials

Polymer and filler used in this study were obtained from commercial sources. BioFlex F2110 was supplied by FKuR Kunststoff GmbH (Willich, Germany) with a weight-average molecular weight of 53 kD, density of 1.27 g/cm^3^, melting temperature (Tm) of 153 °C and MFI of 6 g/10 min (190 °C, 2.16 kg) [28]. Wood flour (mainly coming from beech trees) was supplied by La.So.Le (Percoto, Italy), with a particle size ranging from 300 to 500 μm.

### 3.2. Composites Preparation

BioFlex-wood flour green composites were prepared in an OMC (Saronno, Italy) corotating twin- screw extruder. Mechanically mixed polymer pellet and wood flour with weight ratios of 15% or 30% were poured inside the feeder and processed with a screw rotation speed of 120 rpm. The extruder die temperature was set at 180 °C and the adopted temperature profile was 100–120–140–150–160–170–180 °C. Then, the obtained material was pelletized.

### 3.3. Reprocessing

This stage was carried out by means of a HAAKE (Karlsruhe, Germany) Polylab single screw extruder. Extrusion conditions were: screw rotation speed 60 rpm, temperature profile 120–150–180–175 °C. Reprocessing was carried out both on virgin BioFlex (up to four times) and on filled BioFlex (one time), so as to study the influence or recycling on material properties in the presence of wood flour, or not.

Several sample typologies were prepared, changing fiber content and process conditions. All the samples were prepared by compression molding. Table 8 shows a list of sample typologies prepared.

Prior to any step involving high temperatures, samples were dried at 90 °C for 2 h, in order to prevent the possibility hydrolytic chain scission phenomena.

**Table 8:** Sample code, formulation, and processing conditions.

Sample	Wood Flour Content	Reprocessing
V	None	None
R2	None	2
R4	None	4
V-15	15 wt %	None
V-30	30 wt %	None
R4-15	15 wt %	4
R4-30	30 wt %	4
VP-15	15 wt %	1-after loading
VP-30	30 wt %	1-after loading
RP4-15	15 wt %	4 + 1-after loading
RP4-30	30 wt %	4 + 1-after loading

### 3.4. Specimens Preparation

This was carried out by compression molding using a Carver (Wabash, IN, USA) heated laboratory press. Temperature was set at 180 °C, 10 g of pellets were poured into a custom rectangular mold and held in contact with the hot press plates for 1 min 30 s, then a pressure of 1 bar was applied for 1 min 30 s. Finally, pressure was raised till 6 bars for 3 min, before providing a 3 min cooling step down to 90–100 °C.

### 3.5. Mechanical and Dynamic-Mechanical Characterization

Both tensile and dynamic-mechanical tests were performed on specimens (thickness ≈ 0.5–0.7 mm, width = 10 mm) cut off from the compression molded sheets. In tensile tests, an Instron (Norwood, MA, USA) mod. 3365 universal machine was used, with the crosshead speed set to 5 mm/min for composites testing, while for pure BioFlex it was chosen to operate at 1 mm/min for the first minute and then to accelerate at 10 mm/min, due to the high deformability of the neat material. At least seven measurements were performed for each sample type. Dynamic-mechanical thermal analysis (DMTA) was performed by means of a 01 dB-METRAVIB (Limonest, France) DMA50N apparatus, choosing a heating rate of 5 °C/min up to 120 °C, a strain of 0.05% (after performing an appropriate strain sweep test, in order to remain within the limits of the linear viscoelastic range) and a frequency of 1 Hz.

To evaluate the soundness of the data analysis, statistical analysis was performed through unpaired Student *t*-test, when sets of data were partially overlapped. Differences between two sets of data were considered statistically relevant, and not due to experimental fluctuations, when the *p*-value obtained was lower than 0.05.

Reproducibility of the results was adequate (max. ± 10%).

### 3.6. Viscoelastic Response

Creep tests were performed on all the materials by applying a stress of 1.5 MPa in a dedicated apparatus produced by IDEA (Termini Imerese, Italy). This is basically an oven, equipped with four extensometers, directly connected to mobile clamps and weight holders. The specimens were then mounted between the two clamps and the test started when the weights were applied at the end of the extensometer. The data were thus collected and subsequently elaborated in terms of deformation against time.

### 3.7. Thermal Analysis

Thermal analysis was performed by means of a Perkin Elmer (Waltham, MA, USA) DSC 7 equipment. Samples underwent a first heating from 35 °C up to 190 °C, a cooling step to 35 °C and eventually a second heating step up to 190 °C. The heating and cooling rate was set at 10 °C/min and before each temperature sweep an isothermal step was held for at least 2 min, so as to guarantee thermal homogeneity inside the sample. Enthalpy values found for composites were normalized on the actual amount of polymer involved in the transition, being wood flour not involved in melting and crystallization processes.

### 3.8. Morphological Analysis

The morphology of the fracture surfaces of the prepared systems was investigated by SEM micrographs collected on samples fractured in liquid nitrogen and covered with gold to make them conductive, using a PhenomWorld (Eindhoven, The Netherlands) Phenom ProX scanning electron microscope.

## 4. Conclusions

In this work, green composites from biopolymer matrix (BioFlex) and wood flour have been prepared. Several factors have been investigated, such as the effect of reprocessing on the matrix properties, wood flour loading effects on virgin and reprocessed biopolymer, and wood flour effects on material reprocessability. Tensile, DMTA, DSC, and creep tests pointed out that the addition of wood flour to pristine biopolymer leads to a significant increase of the elastic modulus and creep resistance, without decreasing the tensile strength, which showed some moderate increase. This indicates that good adhesion was obtained between wood fibers and biopolymer matrix, as also demonstrated by SEM analysis. Reprocessing of the biopolymer did not significantly decrease the properties of the pristine one, thus pointing out that this biopolymer can effectively undergo multiple instances of reprocessing. Furthermore, the addition of wood flour to reprocessed biopolymer increased the properties in the same way observed for the pristine. The green composites obtained in this way were subjected to further reprocessing, and it was found that the main properties did not decrease, or even increase, as in the case of the elastic modulus. This gives positive indications regarding the reprocessability of these green composites.
